# The Story of the Insane from Year to Year

**Published:** 1903-02-21

**Authors:** 


### THE STORY OF THE INSANE FROM YEAR TO YEAR.

Fifteenth Annual Series.

### THE JAMAICA ASYLUM.

This is by no means a small asylum, for it contained 8[illegible] patients on April 1st, and on March 31st 863 remained unde[illegible] treatment. There were 158 first admissions, 24 readmission[illegible] 103 discharged recovered, 4 discharged relieved, and 7[illegible] deaths. The average number resident was 862, and the total number under care during the year was 1,050. As might be expected there was great variety in the nationalities and colour of the patients admitted. Seven were white, 51 were brown, 113 were black, 10 were coolies, and one was Chinese. Of the deaths, 31 were caused by cerebral and spinal diseases, 25 by thoracic affections, and 16 by abdominal diseases. Calculated on the admissions, the recoveries were 59·89 per cent., a very high proportion. Calculated on the number under treatment the deaths were 7·43 per cent. Hereditary influence accounts for 32·4 per cent. of the admissions. Alcoholic intemperance is unknown as a cause of insanity in this asylum, and general paralysis is practically absent among the black and coloured population. Dr. Plaxton, the medical superintendent, obtained leave of absence owing to ill health and Dr. Williams was appointed acting superintendent. Dr. Neish was appointed assistant medical officer. The weekly cost per head was 6s. 4d.

